# Small Cell Neuroendocrine Carcinoma of the Urinary Tract Successfully Managed with Neoadjuvant Chemotherapy

**DOI:** 10.1155/2013/598325

**Published:** 2013-08-18

**Authors:** Mustapha Ahsaini, Omar Riyach, Mohammed Fadl Tazi, Mohammed Jamal El Fassi, My Hassan Farih, Hind Elfatmi, Afaf Amarti

**Affiliations:** ^1^Department of Urology, University Hospital Center Hassan II, 30000 Fez, Morocco; ^2^Department of Pathology, University Hospital Center Hassan II, 30000 Fez, Morocco

## Abstract

*Introduction*. Small cell neuroendocrine carcinomas of the urinary tract is an extremely rare entity and very few cases have been reported in the literature. Small cell neuroendocrine carcinoma of the urinary tract (SCC-UT) is the association between bladder and urinary upper tract-small cell carcinoma (UUT-SCC). It characterized by an aggressive clinical course. The prognosis is poor due to local or distant metastases, and usually the muscle of the bladder is invaded. *Case Presentation*. We report a rare case of a 54-year-old Arab male native of moroccan; he is a smoker and was referred to our institution for intermittent hematuria. Following a diagnosis of small cell neuroendocrine carcinomas of the ureter and the bladder, thoracoabdominal-pelvic CT was done, and the staging of the tumor was done in the bladder (T2N0M0) and (T1N0M0) in the ureter. Neoadjuvant alternating doublet chemotherapy with ifosfamide/doxorubicin and etoposide/cisplatin was realized, and nephroureterectomy associated to a cystoprostatectomy was carried out. After 24 months of followup, no local or distant metastasis was detected. *Conclusion*. The purpose of this review is to present a rare case of pure small cell neuroendocrine carcinoma of the urinary tract and review the literature about the place of neoadjuvant chemotherapy in this rare tumors.

## 1. Introduction

Small cell carcinoma (SCC) occurs in the tracheobronchial tree. The extrapulmonary SCC has been described in a variety of organs, including the esophagus, stomach, pancreas, gallbladder, uterine cervix, kidney, urinary bladder, and prostate [1]. Urinary tract small cell carcinoma (UT-SCC) presents a morphology similar to its counterpart in the lung. Most of the described urinary SCC tumors have been reported in the bladder [2]. The primary small cell carcinoma of the urinary tract (UT-SCC) is an extremely rare entity. It is often of high stage at initial diagnosis, with higher metastatic potential and poorer prognosis than pure urothelial carcinomas. The prognosis in patients with UT-SCC tumors remains poor but the improvements in systemic multiagent chemotherapy, especially neoadjuvant chemotherapy with aggressive surgical approach, can improve the long-term survival [2].

In the best of our knowledge, there are a few case reports of UT-SCC located contemporarily in the ureter and the bladder; in the light of our case report, we present a recent systematic literature review regarding the clinical presentation and the management of this rare tumor.

## 2. Case Presentation

**Figure 1:**
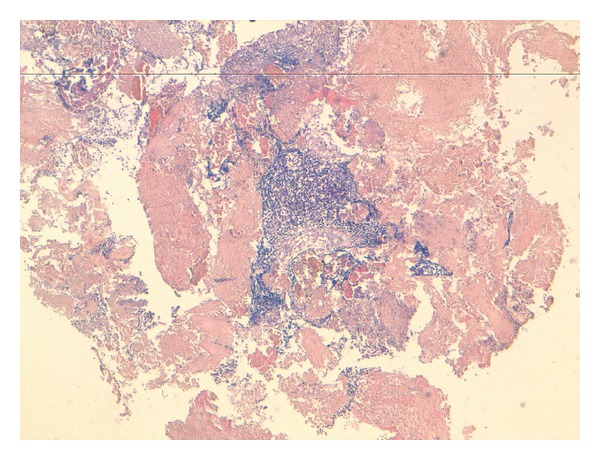
Urothelial submucosa unfiltered by poorly differentiated carcinomatous proliferation comprised sheets of monomorphic cells (HES ×4).

**Figure 2:**
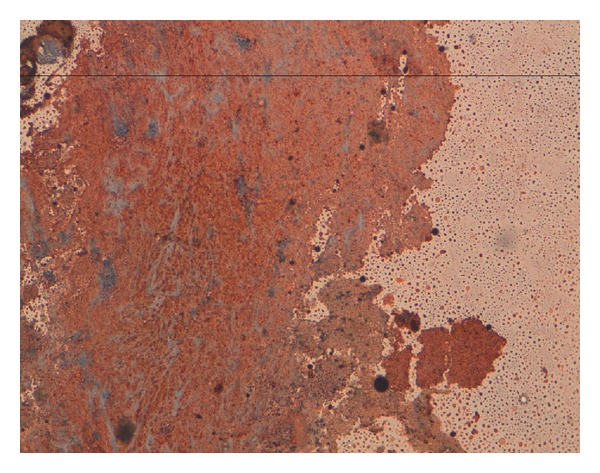
Immunostaining: cytokeratin antibody positive bladder tumor cells.

**Figure 3:**
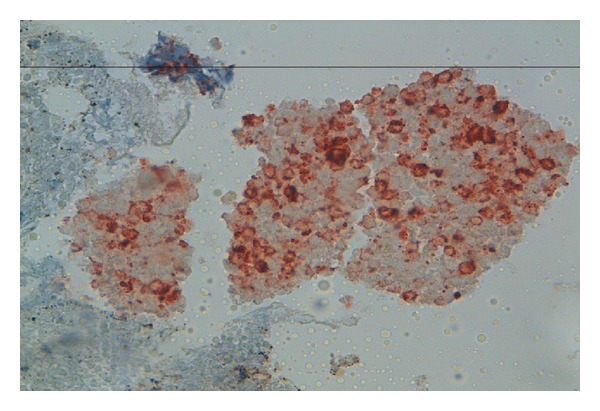
Immunostaining: chromogranin antibody positive bladder tumor cells.

**Figure 4:**
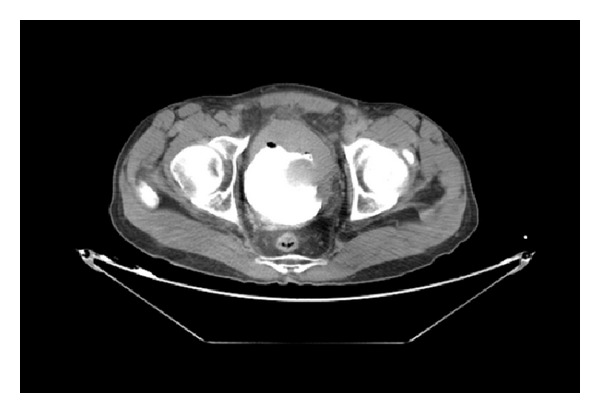
Abdominal-pelvic computerized tomography showed mass in the anterior wall of the urinary bladder.

**Figure 5:**
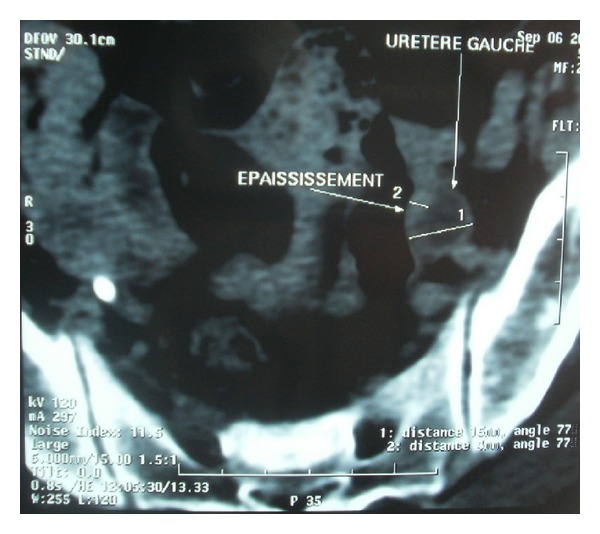
Abdominal-pelvic computerized tomography showed a lesion at the lower one third of the left ureter.

**Figure 6:**
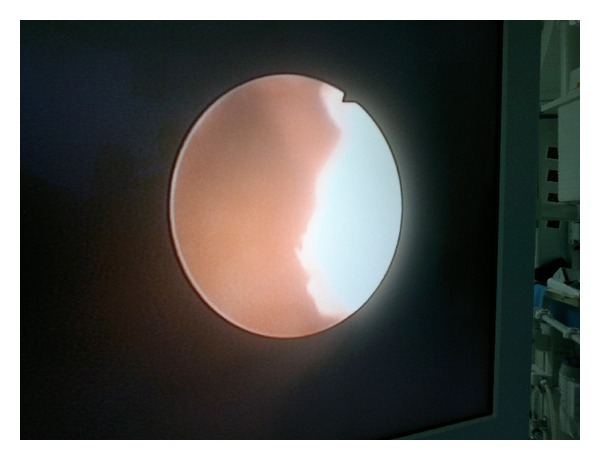
The macroscopic appearance of the tumor in the ureteroscopy.

We report the case of a 54-year-old Arab male Moroccan, without other comorbidities outside smoking, referring to our institution for one month intermittent gross hematuria. Ultrasonography and cystoscopic examination revealed a 4 × 5 cm sessile tumoral mass in the anterior wall of the urinary bladder. Transurethral resection of the tumor mass was performed, and tissue fragments were sent to the pathology department to establish the histological type, the degree of differentiation, and invasion. The cytomorphologic features demonstrated atypical cells with abundant cytoplasm, large nuclei with coarse chromatin, and a high mitotic index (Figure 1). Immunohistochemical staining showed that the tumor components were positive for cytokeratin 7 (Figure 2) and for neuroendocrine markers such as neurone specific enolase (NSE), chromogranin (Figure 3), and synaptophysin. A contrast enhanced abdominal-pelvic computerized tomography (CT) scan (Figure 4) revealed a 4 × 5 cm mass in the anterior wall of the urinary bladder, with a lesion at the lower one third of the left ureter (Figure 5). Flexible ureterorenoscopy was realized, and the diagnosis of SCC of the ureter was done (Figure 6). Preoperative thoracoabdominal-pelvic CT scan was negative for any evidence of local or metastatic disease. Because the optimal management of this tumor is not very well defined, and due to some data in the literature, which report a better prognosis for patients treated with neoadjuvant chemotherapy, and after discussions between oncologist, pathologist, and surgeon, the patient was proposed for neoadjuvant chemotherapy which was doublet chemotherapy consisted of IA alternating with EP for four cycles associated to nephrouretectomy and radical cystoprostatectomy (extensive iliac lymphadenectomy; permanent bilateral ureterostomy was realized).

Our protocol was as follows. Ifosfamide 2,000 mg/m^2^ was infused over 3 hours daily on days 1 through 4. Doxorubicin 25 mg/m^2^ was infused daily on days 1 through 3. Etoposide 80 mg/m^2^ was infused over 2 hours daily on days 1 through 5; cisplatin 20 mg/m^2^ was infused in 1L of normal saline with mannitol 20 g daily on days 1 through 5.

The chemotherapy was well tolerated, and the postoperative course was uneventful. The subsequent surgical specimen confirmed the presence of a pure small cell neuroendocrine tumor of the bladder (pT2) and the distal portion of the ureter (pT1) with lymph nodes negatives for malignancy. Three months after surgery, thoracoabdominal-pelvic CT control was free of local recurrence or distal metastasis. The patient was free of diseases with 2 years of followup.

## 3. Discussion

Neuroendocrine (NE) tumors account for approximately 1% of all primary bladder tumors and include small cell carcinoma, large cell carcinoma, and carcinoid tumor. Pure small cell urinary carcinomas (SCUCs) are the most common kind of NE differentiation in the bladder: they account for 0.48%–1% of all bladder carcinomas, but in reality they are more frequent since they often coexist with conventional urothelial carcinomas. For the upper urinary tract (UUT), the real incidence of UUT-SCC is unknown, and only few cases are reported in the literature [3]. Therefore, the urinary bladder is the most common site of urinary tract SCC, with primary renal pelvis and ureteral SCC occurring extremely rarely [4]. Here, we report a rare association between bladder and UUT-SCC.

After Shahab (2007) [5], risk factors are unknown but there is hypothesis for bladder localizations that these tumors are usually found in smokers, patients affected by long-standing cystitis, those with bladder lithiasis, and those with augmented cystoplasty [5–7]; for the UUT localizations, there is not any hypothesis due to lack of data, but most patients (59%) were of Asian background; this could reflect an increased risk in this population due to the presence of genetic susceptibility or environmental factors [8].

This neoplasm is strongly predominant in males and develops between the fifth and ninth decades of life. The origin of the small cells is still subject to debate, but it is now believed that the small cell carcinoma of the bladder or the UUT originates from the totipotent stem cells present in the submucosa of the urinary tract [5]. There are no specific clinical features differentiating these patients from transitional carcinoma of the bladder if the localization was in the bladder, with the most common symptomatology is hematuria due to hypervascularity and many ulceronecrotic areas, with or without dysuria [9], and from those of renal clear cell carcinoma for the SCC of UUT, with hematuria and flank pain due to hydronephrosis, which being the most commonly reported symptoms, generally when this symptom appear, the stage of tumor might be high. For our patient, he has already hematuria; it is why the disease has a local extension. The tumor is usually located at the lateral and fundus of the bladder, while rarely occurring in the trigone within a bladder diverticulum [10]; it is usually as a polypoid lesions and is sometimes ulcerated [11], but for the UUT-SCC the tumor is two times more likely to be ureteral than pyelocaliceal. This is in contrast to UUT-UC [12]. Urinary tract SCC shares the same histologic features of lung SCC, which are small cells with round to fusiform shape, inconspicuous or absent nucleoli, scanty cytoplasm, and high mitotic activity; the diagnosis depends on histopathological recognition and reactivity for neuroendocrine markers as synaptophysin, chromogranin-A, and neuron-specific enolase [6]. It has already been reported that C-kit, EGFR, BCL2, and CD-56 represent potential targets of molecular therapy and have been identified immunohistochemically in SCC of the prostate [13]. Histologic analysis with immunohistochemistry distinguishes SCC from the differential diagnoses (poorly differentiated UC, plasmacytoid carcinoma, malignant lymphoma, lymphoepithelioma like carcinoma, or primitive neuroectodermal tumor) [14].

Metastasis to the upper urinary tract, originating from a primary SCC of the lung, should be excluded by chest radiography or thoracic computed tomography [15].

These tumors generally show a local extension (T3-T4) in 51%–100% of cases and lymph node or bone, liver, and brain involvement in 28%–80% of patients if the localization was in the bladder [7], and if it was in the UUT, seventy-four percent of patients had nonorgan confined disease (≥pT3) or detectable metastasis at time of diagnosis emphasizing an aggressive behavior and a late clinical presentation during the natural history of these tumors [15]. The prognosis for patients with bladder SCC is poor, with an overall 3-year survival rate of 13%–27%. Such outcome is substantially similar to the prognosis of UUT-SCC with reported 3-year survival rates of 23.8% [7, 16]. Furthermore, pathological stage was the only statistically significant prognosis factor associated with survival for both localizations (bladder, UUT) [2, 17].

Many treatments have been tried, but the optimal management of these tumors has multimodality therapy, which includes surgery (cystoprostatectomy, nephroureterectomy) chemotherapy, and radiation [18]. Some studies showed that cisplatin-based chemotherapy appears to have a better survival and prognosis [5, 19]. Many authors recommend platinum-containing and etoposide regimens, especially for extra bladder invasion [10, 20, 21]. Some data in the literature report a better prognosis for patients treated with a chemotherapy regimen preoperatively [2, 22–24].

Without a large randomized trial, it is impossible to confirm the benefits of neoadjuvant chemotherapy; however, the improved clinical outcomes and high likelihood of pathologic downstaging seen in the current clinical trial support the utility of neoadjuvant chemotherapy.

## 4. Conclusion

Small cell carcinoma of the urinary tract is a distinct histological and biologic disease entity with an aggressive clinical course, poor prognosis, and average life expectancy of only few months. Therapeutic modalities vary from institution to institution.

This case report is consistent with previously reported retrospective data demonstrating long-term survival with four cycles of neoadjuvant chemotherapy for surgically resectable SCUC.

Improvements are expected in the future in terms of effective systemic therapies focusing on the role of targeted therapies.
